# Serotoninergic modulation in the brainstem and hypothalamus of female overnourished rats: impact on mitochondrial markers, oxidative stress and BDNF mRNA levels

**DOI:** 10.3389/fmolb.2025.1564061

**Published:** 2025-05-16

**Authors:** Thyago de Oliveira Rodrigues, Osmar Henrique dos Santos Júnior, Maria Daniele Teixeira Beltrão de Lemos, Matheus Santos de Sousa Fernandes, Fatma Hilal Yagin, Burak Yagin, Samarjit Das, Abdullah F. Alghannam, Pablo Prieto-González, Claudia J. Lagranha

**Affiliations:** ^1^ Graduate Program in Nutrition, Physical Activity and Phenotypic Plasticity, Academic Center of Vitória, UFPE - CAV, Federal University of Pernambuco, Recife, Brazil; ^2^ Graduate Program in Neuropsychiatry and Behavioral Sciences, Federal University of Pernambuco, Recife, Brazil; ^3^ Keizo Asami Institute, iLIKA - UFPE, Federal University of Pernambuco, Recife, Brazil; ^4^ Department of Biostatistics, Faculty of Medicine, Malatya Turgut Özal University, Malatya, Türkiye; ^5^ Department of Biostatistics and Medical Informatics, Faculty of Medicine, Inonu University, Malatya, Türkiye; ^6^ Department of Anesthesiology and Critical Care Medicine, Johns Hopkins School of Medicine Baltimore, Baltimore, MD, United States; ^7^ Lifestyle and Health Research Center, Health Sciences Research Center, Princess Nourah bint Abdulrahman University, Riyadh, Saudi Arabia; ^8^ GSD-HPE Department, Sport Sciences and Diagnostics Research Group, Prince Sultan University, Riyadh, Saudi Arabia

**Keywords:** obesity, serotonin, fluoxetine, hypothalamus, overnutrition, oxidative stress

## Abstract

**Introduction:**

Obesity is a global epidemic identified by the World Health Organization, and its complexity involves genetic, cultural, socioeconomic, and behavioral factors.

**Methods:**

In this study, we used female Wistar rats, with litters standardized to nine female pups, which were divided into two groups: normally nourished or overnourished. The groups were further subdivided into control and fluoxetine-treated groups, with the pharmacological treatment maintained until the 21st day of life. At 30 days of age, euthanasia was performed, and tissues from the hypothalamus and brainstem were collected.

**Results:**

We observed an increase in body weight and the Lee index in the overnourished group, but fluoxetine treatment reduced these indices. Additionally, overnourished rats consumed more palatable food. Biochemically, NADH content in the hypothalamus was altered by overnutrition but restored by fluoxetine treatment. Citrate synthase activity was reduced in the overnourished group in the hypothalamus but increased in the brainstem of fluoxetine-treated rats. The production of reactive oxygen species was higher in the overnourished group, and oxidative stress biomarkers showed increased levels of MDA and protein carbonylation in these rats. Overnutrition impaired the antioxidant activity of enzymes in both the hypothalamus and brainstem, whereas fluoxetine treatment improved this activity. BDNF expression was higher in the fluoxetine-treated groups compared to the overnourished group.

**Discussion:**

These results demonstrate the detrimental effects of maternal overnutrition on the development of female offspring and the therapeutic potential of serotonergic manipulation to mitigate the early effects of obesity, with tissue-specific variations.

## 1 Introduction

Imbalances in nutrition during critical developmental periods can exert profound and enduring effects on the offspring’s metabolism, potentially predisposes them to obesity and associated metabolic disorders later in life (Rajamoorthi et al., 2022). This phenomenon, termed developmental programming, occurs when environmental factors, such as maternal nutrition, influence the development of the fetus’ organs and systems, leading to persistent structural and functional alterations (Ludwig et al., 2023). These changes encompass modifications in hormone levels, metabolic pathways, and the expression of genes involved in energy balance and fat storage, thereby shaping the individual’s physiological trajectory (Gluckman et al., 2005). Previously, studies demonstrated that nutritional stress during an early critical period of development in rats can disrupt the fine control of food intake and lead to hyperphagia and obesity (da Silva et al., 2019; Braz et al., 2020a). Studies with overnutrition demonstrated an increase in white adipose and a decrease in brown adipose tissue, deregulation of food intake, increased blood levels of glucose and triglycerides, and modification of hypothalamic NPY and CART gene expression in early adulthood (da Silva et al., 2019; de Andrade Silva et al., 2021). The generalized hypothesis used to explain such observations is that nutritional insults in early life may result in a predictive adaptive response of the developing organism designed for immediate survival in an adverse environment, which may result in obesity (Gluckman et al., 2005; Nettle et al., 2013).

The burgeoning prevalence of childhood obesity, as highlighted by the World Health Organization’s statistics indicating approximately 39 million overweight or obese children under the age of five in 2020, underscores the urgency of understanding the multifaceted etiology and consequences of this condition (WHO, 2021). Beyond its immediate impact on physical health, childhood obesity can profoundly affect social, emotional, and psychological wellbeing, with long-term ramifications extending into adulthood (Sahoo et al., 2015). Moreover, mounting evidence suggests a concerning association between childhood obesity and heightened morbidity and premature mortality risks (Tiwari and Balasundaram, 2023). The etiology of obesity is intricate, reflecting an interplay of genetic, hormonal, environmental, lifestyle, and emotional determinants. This complexity is further underscored by the global escalation in obesity prevalence, positioning overweight and obesity as the fifth leading risk factor for global mortality (Mamdouh et al., 2023). At a molecular level, investigations into childhood obesity have unveiled a spectrum of alterations encompassing epigenetic modifications, insulin resistance, elevated inflammatory markers like C-reactive protein (CRP), and dysregulation of the gut microbiome, collectively impinging upon lipid and glucose homeostasis as well as satiety signaling pathways (Orsso et al., 2021; Marcus et al., 2022).

Chronic low-grade inflammation represents a hallmark of obesity, precipitating a persistent elevation in oxidative stress levels. This oxidative milieu poses a threat to cellular integrity. It has been implicated in the pathogenesis of various neurological conditions, including Parkinson’s disease, Alzheimer’s disease, amyotrophic lateral sclerosis (ALS), multiple sclerosis, depression, and cognitive decline (Pizzino et al., 2017; Marseglia et al., 2015). Notably, the extent of oxidative damage exhibits sexual dimorphism, with women benefiting from inherent antioxidant protection conferred by estrogen, the primary female sex hormone governing reproductive and physiological processes (Borrás et al., 2010; Iorga et al., 2017; Lagranha et al., 2018). However, despite estrogen’s antioxidative properties, its protective efficacy appears attenuated in obesity, where chronic inflammation, insulin resistance, endothelial dysfunction, and impaired cognitive function prevail (Kwaifa et al., 2020; Sui and Pasco, 2020). Moreover, pharmacological agents may disrupt estrogen levels and signaling in clinical scenarios, compromising its protective effects (Domingues et al., 2023). This convergence of factors challenges the mitigating influence of estrogen in the face of oxidative stress and inflammation associated with obesity, warranting further investigation into the intricate interplay between sexual dimorphism, oxidative balance, and obesity-related pathophysiology.

Against this backdrop, the present study endeavors to elucidate the impact of maternal overnutrition on body weight, eating behavior, blood profile, oxidative balance, mitochondrial function, and the expression of genes implicated in neural plasticity within the hypothalamus and brainstem of female rats exposed to overnutrition during the critical developmental window extending up to 30 days postnatally. It is worth noting that the focus on female offspring is based on the growing recognition of sex-specific differences in metabolic regulation, hormonal responses and susceptibility to obesity-related pathologies. Emerging evidence suggests that females exhibit distinct neuroendocrine adaptations to metabolic stress, and their physiological response to early nutritional insults may differ markedly from that of males. Through a comprehensive examination of these neurobiological milieus, this research aims to shed light on the intricate mechanisms underpinning the metabolic disturbances and inform targeted interventions to mitigate the burgeoning burden of obesity and its attendant complications.

## 2 Materials and methods

### 2.1 Animals and experimental design

All procedures in the experiment complied with the ethical guidelines set forth by the Animal Research Ethics Committee (approval protocol no 0013/2023) and the “Principles of Laboratory Animal Care” published by the National Institutes of Health, Bethesda, MD, United States.

Ten female Wistar rats, aged 80 days and weighing between 150 and 200 g, were mated with five male rats, aged 120 days, and weighing between 200 and 250 g. The animals were kept at 23°C ± 1°C with a 12-h light-dark cycle and provided free access to water and commercial feed (Labina-Presence®) during gestation and lactation. Twenty-four hours after birth, the female pups (weighing 6–8 g) were randomly assigned to groups, with nine pups per mother. On the third postnatal day (PND 3), the groups were divided to adjust litter size: the normofed group remained with nine female pups per litter, while a super-fed group was created with three female pups per litter. On the third day of life, we started treatment on the female rat pups, who were subdivided into four groups, namely Normo saline (NS), Normo fx (NF), Over saline (OS), Over fx (OF). The reduced litter size increased the offspring’s food availability, resulting in increased food intake and, consequently, higher body weight. At PND 21, all females were weaned and housed in cages with five animals per cage, receiving water and commercial feed *ad libitum* until they reached 30 days of age.

### 2.2 Pharmacological treatment, experimental groups, and body weight assessment

From the third postnatal day (PND3) to the twenty-first postnatal day (PND21), the rats were given a daily subcutaneous injection of either saline solution (NaCl: 0.9%, 10 mL/kg body weight) or fluoxetine, a selective serotonin reuptake inhibitor (SSRI) (10 mg/kg body weight, vehicle solution, 10 mL/kg body weight). The concentration used in this study was determined in previous studies to ensure no side effects on mitochondrial and oxidative balance (da Silva et al., 2019). The rats were divided into four groups based on their body weight and weight gain from the beginning of the study: Normofed + saline solution (Normofed), Normofed + fluoxetine (Normofed + Fx), Overfed + saline solution (Overfed), and Overfed + fluoxetine (Overfed + Fx). The pharmacological treatment was always administered in the second hour after the start of the dark cycle to avoid potential influences on the circadian rhythm. Body weight (g) was measured once a week during the lactation period until the rats were weaned at 21 postnatal days, using a digital scale with an accuracy of 0.1 g.

### 2.3 Food consumption and palatable preference

Food intake was measured by weighing the remaining commercial feed after 12 h of light and 12 h of darkness from 25 to 29 days of age. After 3 h of food deprivation, this was done using a digital balance (Mars, model S-100 with 0.001 g sensitivity). The results were expressed in grams. For the food preference experiments performed at 22 days, animals were allowed to adapt to the cage and diet for 1 day, and the measurements occurs during three consecutive days. At both 22 days, rats were allocated to individual cages and given 50 g of palatable food (chocolate cookies-Chocookies; Nabisco®, East Hanover, NJ, United States), according previous published (de Andrade Silva et al., 2021).

### 2.4 Lee index

The Lee index (Lee, 1929) is a practical and efficient method commonly used to assess obesity in rodents subjected to weight gain protocols. It is calculated by dividing the cube root of the animal’s body weight (in grams) by its naso-anal length (in millimeters) and then multiplying the result by 1,000. Values below 0.300 are generally considered to fall within the normal range. Research has shown that the Lee index correlates well with body fat mass, making it a reliable indicator of adiposity and overall nutritional status in experimental models involving rodents.

### 2.5 Blood collection and serum analysis

Blood samples were collected on the day of euthanasia and stored in a tube. The samples were centrifuged at 3,500 RPM in 10 min to extract the serum. The supernatant was used for biochemical analysis using Labtest® colorimetric kits to assess glycemic levels, total cholesterol, HDL, and triglycerides.

### 2.6 Sample preparation for oxidative stress analysis

At 30 days of age, the rats were sacrificed by decapitation using commercially available guillotine-type equipment, and the brain regions containing the brainstem and hypothalamus were quickly dissected and preserved at −80°C for further analysis. For the biochemical experiments, the tissues were homogenized in Tris-EDTA buffer (Tris 100 mmol·L–1, pH 7.5; EDTA 10 mmol·L–1, NP40 0.1%, and protease inhibitors) on ice and centrifuged for 10 min at 5,000 × g at 4°C. Aliquots of the supernatant were analyzed for total protein content using the Bradford protocol (Bradford, 1976). The tissues for RT-PCR assays were kept at −80°C until adequately prepared.

### 2.7 Pyridine nucleotide oxidation kinetics (NAD+/NADH ratio)

NAD+ and NADH levels were determined as previously described (Lehninger, 1971). 100μg of protein samples were incubated with a buffer containing 50 mM-TRIS, and 1 mM-EDTA (pH 7.4) was measured using a spectrophotometer (IL-592, ELEVE, China) at room temperature for 1 min. Then, the absorbance was read at two wavelengths, 260 nm, and 340 nm, for NAD+ and NADH, respectively, to evaluate pyridine nucleotide oxidation kinetics (NAD+/NADH ratio).

### 2.8 Citrate synthase enzyme activity

To verify oxidative capacity and mitochondrial function, we focused on the first enzyme of the Krebs cycle, citrate synthase. This enzyme, known for its reliability as an indicator of the system’s functionality, plays a pivotal role in the cycle. The activity was assessed using a mixture containing Tris-HCl (pH = 8.2), magnesium chloride (MgCl), ethylenediaminetetraacetic acid (EDTA), 0.2 mM of 5.5 dithiobis (2-nitrobenzoic acid), 3 mM acetyl-CoA, 5 mM oxaloacetate, and 0.3 mg/mL of homogenized tissue. The reaction was carried out at a temperature of 25°C, and the activity was measured by assessing the change in absorbance at 412 nm for 3 min. The levels are expressed as U/mg of protein. This protocol was conducted as previously described (Le Page et al., 2009).

### 2.9 Evaluation of reactive oxygen species (ROS) production

In this experiment, we employed a robust method, known for its accuracy and sensitivity, to evaluate reactive oxygen species (ROS) production. Samples (200 µg) were incubated in TRIS-HCL buffer (40 mM) at pH 7.4 with 2′,7′-DCFH-DA (10 µM) for 30 min at 30°C. This method allows for accurate and sensitive detection of ROS production. After incubation, the samples’ fluorescence was measured on a Varioskan microplate reader (ThermoFisher, United States) at 485 nm excitation and 525 nm emission. The results were expressed as fluorescence intensity per mg of protein (da Silva et al., 2019).

### 2.10 Evaluation of malondialdehyde production

To measure the product malondialdehyde (MDA), 200 μg of protein was used and reacted with thiobarbituric acid (TBA). MDA or MDA-like substances produce a pink pigment with maximum absorption at 535 nm. The reaction was initiated by sequentially adding 30% trichloroacetic acid (TCA) and Tris-HCl (3 mM) to the samples, followed by centrifugation at 2,500 *g* for 10 min. The supernatant was transferred to a tube, mixed with an equal volume of 0.8% (v/v) TBA, and boiled for 30 min. The absorbance of the organic phase was read at 535 nm in a spectrophotometer. The results were expressed in mmol per milligram of protein (Buege and Aust, 1978).

### 2.11 Evaluation of protein oxidation

The carbonyl content in the protein was measured to measure oxidative damage to the protein. For this, 30% TCA was added to the sample on ice, mixed, and centrifuged for 15 min at 1,180×g. The sediment was suspended in 2,4-dinitrophenylhydrazine (DNPH) 10 mM and incubated immediately in the dark for 1 h with agitation every 15 min. The samples were then centrifuged and washed three times with the ethyl/acetate buffer, and the pellet was suspended in 6 M guanidine hydrochloride. It was then incubated for 5 min in a water bath at 30°C. The absorbance was read at 370 nm, and the results were expressed in mmol per milligram of protein (Levine et al., 1990).

### 2.12 Measurement of superoxide dismutase (SOD) activity

The total activity of the superoxide dismutase enzyme (t-SOD) was measured. For this, supernatants (0.2 mg/mL) were incubated with 880 μL of sodium carbonate (0.05%, pH 10.2, 0.1 mM EDTA) at 25°C, and the reaction was initiated with 30 mM epinephrine (in 0.05% acetic acid). The kinetics of inhibition of adrenaline autoxidation were monitored for 180 s at 480 nm. The result was expressed in units per mg of protein (Misra and Fridovich, 1972).

### 2.13 Measurement of catalase (CAT) activity

The CAT activity was measured in the assay consisting of 50 mM phosphate buffer (pH 7.0), 0.300 mM H_2_O_2_, and a sample (200 μg of protein). The enzyme velocity constant was determined by measuring the change in absorbance at 240 nm for 4 min at 25°C. The CAT activity was expressed in units per mg of protein (Aebi, 1984).

### 2.14 Measurement of glutathione-S-transferase (GST) activity

For this experiment, the GST activity was measured in 200 μg of the supernatant added to 0.1 M phosphate buffer (pH 6.5) containing 1 mM EDTA at 25°C. The assay was initiated with 1 mM 1-chloro-2,4-dinitrobenzene plus 1 mM GSH. The formation of 2,4-dinitrophenyl-S-glutathione was monitored at 340 nm absorbance, and the enzymatic activity was defined as the amount of protein needed to catalyze the formation of 1 μmol of 2,4-dinitrophenyl S-glutathione. The results were expressed in units per mg of protein (Habig et al., 1974).

### 2.15 Measurement of total thiol (SH) groups

The quantification of thiols was based on the reduction of 5,5′-dithio-bis (2-nitrobenzoic acid) (DTNB) by thiols. To summarize, 200 μg of protein supernatant was mixed with 30 μL of 10 mM DTNB and incubated in the dark. After that, an extraction buffer (pH 7.4) was added to obtain a final volume of 1 mL. The absorbance was measured at 412 nm, and the results were expressed in mol/mg of protein (Aksenov and Markesbery, 2001).

### 2.16 Measurement of the GSH/GSSG ratio

The Reduced Glutathione/Oxidized Glutathione (GSH/GSSG) ratio was evaluated using a method previously described by Hissin and Hilf (1976). The samples were incubated in 0.1 M phosphate buffer containing 5 mM EDTA (pH 8.0) and with 1 μg/mL o-phthalaldehyde (OPT) at room temperature for 15 min and evaluated by fluorescence with wavelengths of 350 nm and 420 nm. The GSSG levels were evaluated separately by incubating the same samples with 40 mM N-ethylmaleimide for 30 min at room temperature with the addition of 100 mM NaOH buffer. The GSH/GSSG ratio reflects the REDOX state (Hissin and Hilf, 1976).

### 2.17 Preparation for mRNA expression

Total RNA was extracted from the tissues with Trizol reagent using the guanidine isothiocyanate method (Chomczynski and Sacchi, 1987), according to the manufacturer’s instructions (Invitrogen, Carlsbad, CA, United States). The RNA pellets were washed in 75% ethanol, centrifuged at 7,500 *g* for 5 min at 4°C, air-dried, and dissolved in DEPC-treated ultrapure water. RNA quantification was performed on a NanoDrop spectrophotometer 2000c (Thermo Scientific, United States), and purity was assessed using the absorbance ratio of 260/280 nm. Then, real-time polymerase chain reaction (RT-PCR) reactions were performed for β2-microglobulin (β2M), brain-derived neurotrophic factor (BDNF) (Table 1) according manufacture protocol for the SuperScript® III Platinum® SYBR® Green qRT-PCR Kit (Invitrogen, United States). The samples were processed in duplicate, and each target gene’s threshold cycle (Ct) values were normalized to the β2M Ct determined in the same samples. The relative mRNA expression was determined using the 2^−ΔΔCT^ method (Livak and Schmittgen, 2001).

**TABLE 1:** Primer sequences used in this study for the β2M and BDNF genes, including forward and reverse primers in the 5′–3′ direction. The sample size was 4 animals per group.

Primers sequence		
*Gene*	*Forward primer (5’ – 3′)*	*Reverse primer (5’ – 3′)*
β2M	TGACCGTGATCTTTCTGGTG	ACTTGAATTTGGGGAGTTTTCTG
BDNF	ACGGTCACAGTCCTTGAAAG	GGATTGCACTTGGTTCCGTA

### 2.18 Statistical analysis

The data was analyzed using Excel 2010 (Washington, United States) and GraphPad Prism 10 (GraphPad, La Jolla, CA, United States). Since the number of animals in the groups was below 10, the data did not meet parametric assumptions. The data were then expressed as medians with their respective minimum and maximum scores. To compare the experimental groups, an analysis of variance was used using the Kruska-Wallis test with multiple comparisons and Dunn’s post-test. The significance of the comparisons was p < 0.05 (5%).

## 3 Results

### 3.1 Body weight

The body weight of the female rats at 30 days of age was higher in the group of overnourished female rats than in the group of normo-nourished female rats NS: 78.50 (73.00–85.00) and OS: 102.0 (90.00–102.0) with statistical significance (p = 0.047) (Table 2).

**TABLE 2:** Body weight at 30 days of age, Lee index, and Serum analysis from female rats normo- or overfed during the lactation period, treated or not with fluoxetine. The data were then expressed as medians, minimum, and maximum scores.

Variables	Normo + Sal	Over + Sal	Normo + FX	Over + FX
Body weight (g)	78.50 (73.00–85.00)	102.0 (90.00–102.00)*	63.00 (60.00–64.00)	79.00 (75.00–86.00)
Lee index (g/cm^3^)	288.0 (250.0–299.0)	313.0 (300.0–338.0)*	290.0 (249.0–388.0)	291.5 (285.0–295.0)^&^
Glucose (mg/dL)	166.5 (160.2–175.8)	191.5 (187.3–229.7)	165.7 (139.4–184.3)	148.7 (135.6–154.2)^&^
Total cholesterol (mg/dL)	68.56 (67.42–72.52)	81.02 (75.35–88.39)	65.16 (62.32–66.86)	56.66 (46.46–60.06)^&^
HDL (mg/dL)	95.82 (75.23–97.87)	94.79 (92.72–99.68)	84.08 (79.66–92.88)	78.98 (76.15–81.47)
Triglycerides (mg/dL)	73.33 (55.56–78.89)	72.22 (51.11–87.78)	66.67 (60.00–88.89)	66.67 (63.33–73.33)

To compare the experimental groups, an analysis of variance was used using the Kruska-Wallis test with multiple comparisons and Dunn’s post-test. For body weight, the p was *p < 0.047 vs. NS; For Lee’s index was *p < 0.001 vs. NS and &p < 0.021 vs. OF; Glucose &p < 0.0007 vs. OF; Total cholesterol &p < 0.0004 vs. OF; For HDL and Triglycerides, the results not significant (ns). The sample size was 8 animals per group.

### 3.2 Lee’s index

The Lee index indicates obesity in rats, previously described by Lee (1928). In this study, the overnourished female rats were also considered obese because, when comparing the females in the NS and OS groups, the latter had higher index values NS: 288.0 (250.0–299.0), OS: 313.0 (300.0–338.0), with a statistical difference of (p = 0.001). In addition, treatment with fluoxetine reduced the index values in the OF group of female rats when compared to the OS group females, OS: 313.0 (300.0–338.0), OF: 291.5 (285.0–295.0), with a significance of (p = 0.021) (Table 2).

### 3.3 Eating behavior

**FIGURE 1:**
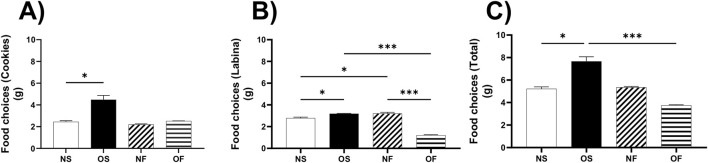
Eating behavior on female rats normo- or overfed during lactation period, treated of not with fluoxetine. **(A)** Food choices (Cookies); **(B)** Food choices (Labina); **(C)** Food choices (Total intake). The data distribution was checked using the Shapiro-Wilk test. The data were then expressed as medians, minimum and maximum scores. To compare the experimental groups, an analysis of variance was used using the Kruska-Wallis test with multiple comparisons and Dunn’s post-test. *p ≤ 0.05, **p ≤ 0.01, p ≤ 0.001, ****p ≤ 0.0001. The sample size was nine animals per group.

Our research revealed a significant preference for more palatable foods, such as cookies, in the female rats of the OS group. They consumed a higher quantity of cookies compared to the NS group, with a value of NS: 2.50 (2.00–3.00), OS: 3.91 (3.83–5.70, p = 0.013, Figure 1A), underscoring the importance of palatability in their eating behavior.

In addition, female rats in the OS group ate more labina, a less palatable food, than female rats in the NS group, NS: 2.88 (2.00–2.90), OS: 3.20 (3.00–3.30), with significance (p = 0.01). The females in the OS group also ate more than the female OF rats, OS: 3.20 (3.00–3.30) and OF: 1.20 (1.00–1.40), with a significance of (p = 0.0002). Interestingly, the female rats in the NF group ate more labina than the NS rats, NS: 2.88 (2.00–2.90) and NF: 3.11 (3.00–4.00). Finally, the females in the NF group ate more than the OF group of females, NF: 3.11 (3.00–4.00) and OF: 1.20 (1.00–1.40), the significance was respectively (p = 0.046 and p = 0.0004) (Figure 1B).

In terms of total consumption between cookies and labina, the overnourished female rats in the OS group ate more than the female rats in the NS group, with NS: 5.38 (4.00–5.88) and OS: 7.03 (7.00–9.00). In addition, the OS female rats also ate more than the OF female rats, thus OS: 7.03 (7.00–9.00) and OF: 3.70 (3.70–3.90), with significance (p = 0.045; p = 0.0001), respectively (Figure 1C).

### 3.4 Biochemical blood tests

Serum glucose levels were higher in female rats in the OS group when compared to the OF group of female rats, with OS: 191.5 (187.3–229.7) and OF: 148.7 (135.6–154.2), with significance (p = 0.0007). Triglyceride and HDL levels did not differ between the groups. Finally, total cholesterol levels were higher in the OS rats than in the OF rats, with OS: 81.02 (75.35–88.39) and OF: 56.66 (46.46–60.06), the significance being (p = 0.0004) (Table 2).

### 3.5 The cellular and mitochondrial function of the hypothalamus and brainstem

**FIGURE 2:**
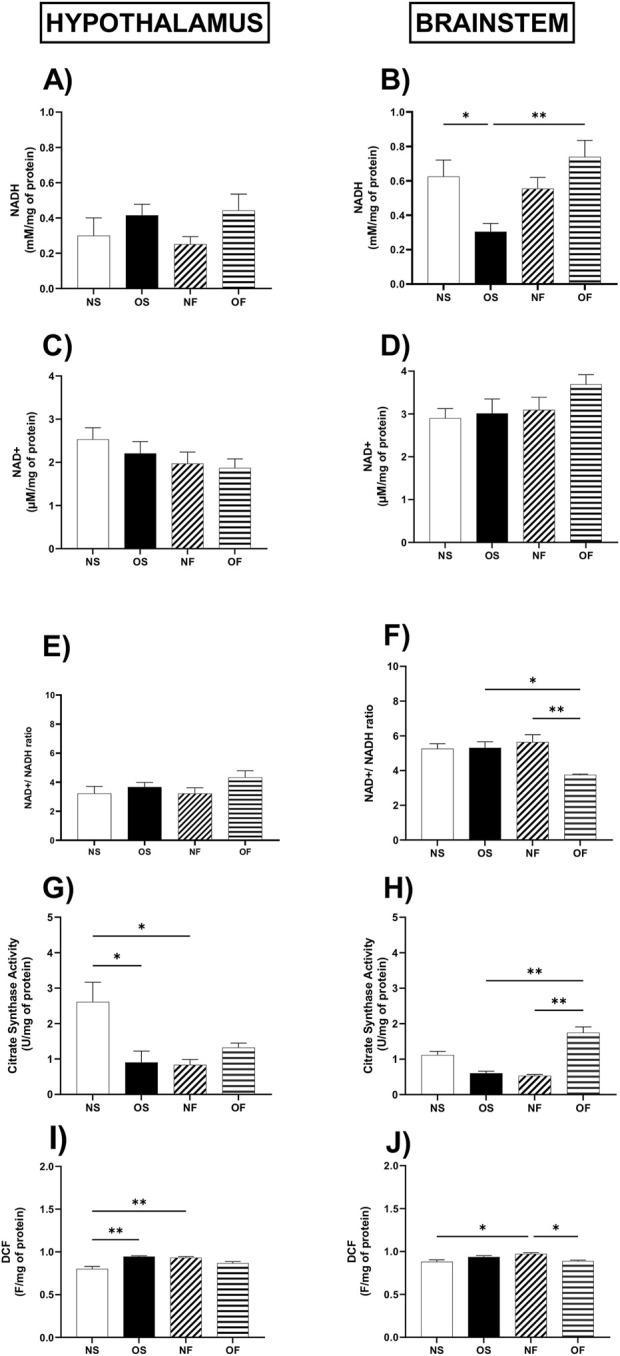
Cellular and Mitochondrial function in the hypothalamus and brainstem from female rats normo- or overfed during lactation period, treated of not with fluoxetine **(A)** NADH in the hypothalamus; **(B)** NADH in the brainstem; **(C)** NAD+ in the hypothalamus; **(D)** NAD+ in the brainstem. **(E)** NAD+/NADH ratio in the hypothalamus; **(F)** NAD+/NADH ratio in the brainstem; **(G)** citrate synthase activity in the hypothalamus; **(H)** citrate synthase activity in the brainstem; **(I)** ROS levels in the hypothalamus; **(J)** ROS levels in the brainstem. The data were then expressed as medians, minimum and maximum scores. To compare the experimental groups, an analysis of variance was used using the Kruska-Wallis test with multiple comparisons and Dunn’s post-test. *p ≤ 0.05, **p ≤ 0.01, p ≤ 0.001, ****p ≤ 0.0001. The sample size was 8 animals per group.

Regarding the evaluation of coenzymes in the reduced and oxidized states, the content of NADH in the hypothalamus did not differ between the groups (Figure 2A); however, in the brainstem, the content of this coenzyme was affected by overnutrition in the OS female rats than in the NS female rats, with NS: 0.49 (0.40–1.33) and OS: 0.31 (0.08–0.49). Despite the overnutrition, the female rats treated with fluoxetine were able to show increased levels of NADH compared to the OS group, thus OS: 0.31 (0.08–0.49) and OF: 0.80 (0.40–1.12), the significance was, respectively, (p = 0.032; p = 0.004) (Figure 2B). The NAD + content did not differ between the groups in any of the tissues evaluated (Figures 2C,D). The NAD+/NADH ratio in the hypothalamus showed no difference (Figure 2E); however, in the brainstem this ratio was higher in the group of OS female rats than OF, thus OS: 4.92 (4.36–8.00) and OF: 3.82 (3. 57–3.83), with significance (p = 0.017), it was also higher in the NF female rats than in the OF group females, thus NF: 5.66 (4.05–7.14) and OF: 3.82 (3.57–3.83), with significance (p = 0.006) (Figure 2F).

The activity of the citrate synthase enzyme showed lower activity in the hypothalamus of OS overnourished female rats compared to NS female rats, thus NS: 2.89 (1.13–3.49) and OS: 0.68 (0.19–2.08), the significance of (p = 0.048). In addition, NF female rats showed a reduction in the activity of this enzyme compared to the NS group of female rats, with NS: 2.89 (1.13–3.49) and NF: 0.91 (0.42–1.30), with significance (p = 0.036) (Figure 2G). In the brainstem, citrate enzyme activity was lower in NF female rats compared to NS female rats; thus, NS: 1.17 (0.82–1.50) and NF: 0.52 (0.44–0.63), the significance was (p = 0.025). About serotonergic manipulation, this treatment increased the activity of this enzyme in female OF rats compared to NF, thus the NF group: 0.52 (0.44–0.63) and OF: 1.65 (1.29–1. 83), also concerning female rats in the OS group, thus OS: 0.69 (0.33–0.76) and OF: 1.65 (1.29–1.83), with significance, respectively, (p = 0.002; p = 0.012) (Figure 2H).

Regarding the production of total reactive species in the hypothalamus (Figure 2I), the female rats in the OS group produced more reactive species than the NS rats, NS: 0.801 (0.708–0.860) and OS: 0.932 (0.887–0.946) (p = 0.003). In addition, NF female rats produced more of these molecules than NS female rats, NS: 0.801 (0.708–0.860) and NF: 0.945 (0.924–0.959), with significance (p = 0.004). In the brainstem, NF female rats showed higher levels of reactive species than NS female rats, NS: 0.879 (0.827–0.929) and NF: 0.936 (0.914–0.985), (p = 0.011). They also showed higher levels than female rats in the OF group, NF: 0.936 (0.914–0.985) and OF: 0.886 (0.865–0.918), with significance (p = 0.019) (Figure 2J).

### 3.6 Oxidative stress biomarkers in the hypothalamus and brainstem

**FIGURE 3:**
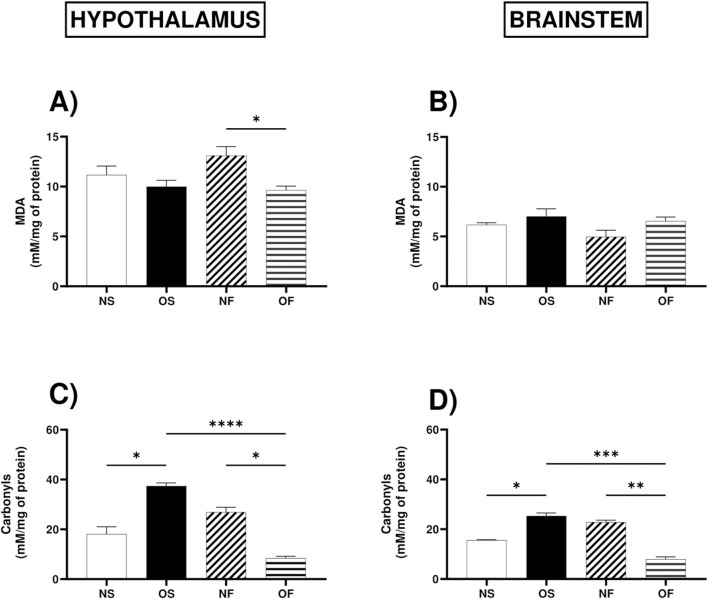
Oxidative stress biomarkers in the hypothalamus and brainstem from female rats normo- or overfed during lactation period, treated of not with fluoxetine. **(A)** MDA in the hypothalamus; **(B)** MDA in the brainstem; **(C)** Carbonyls in the hypothalamus; **(D)** Carbonyls in the brainstem; The data were then expressed as medians, minimum and maximum scores. To compare the experimental groups, an analysis of variance was used using the Kruska-Wallis test with multiple comparisons and Dunn’s post-test. *p ≤ 0.05, **p ≤ 0.01, p ≤ 0.001, ****p ≤ 0.0001. The sample size was 6 animals per group.

About oxidative stress biomarkers, levels of malondialdehyde, a marker of lipid peroxidation, were higher in the hypothalamus of NF female rats, although serotonergic manipulation managed to reduce these levels in OF rats, thus NF: 13.92 (10.33–15.33) and OF: 9.61 (8.46–12.18), with significance (p = 0.026) (Figure 3A). However, in the brainstem, there was no difference between the groups (Figure 3B).

As for the levels of protein oxidation in the hypothalamus, the female rats in the OS group showed increased levels of protein oxidation than the NS female rats, thus NS: 13.94 (12.73–25.45) and OS: 37.12 (33.33–42.12), with significance (p = 0.044). The group of NF rats also showed increased levels of damage, but serotonergic manipulation in the overnourished rats reduced this damage, with NF: 28.94 (17.58–42.12) and OF: 8.48 (6.06–10.91), (p = 0.0.041). In addition, overnutrition in OS female rats increased protein oxidation, however serotonergic manipulation in this group reduced this damage, with OS: 37.12 (33.33–42.12) and OF: 8.48 (6.06–10.91) (p = 0.0001) (Figure 3C). In the brainstem, the response to overnutrition was similar in female rats, where the OS group showed increased levels of protein damage compared to the NS group, NS: 15.82 (14.55–16.18) and OS: 25.09 (22.36–30.91), (p = 0.042), also showed increased levels than OF female rats, thus OS: 25. 09 (22.36–30.91) and OF: 7.90 (5.82–11.27), (p = 0.0002), the NF group also showed increased levels of protein oxidation compared to the OF female rats, thus NF: 22.91 (20.55–24.91) and OF: 7.90 (5.82–11.27), with significance (p = 0.008) (Figure 3D).

### 3.7 Enzymatic antioxidant system in the hypothalamus and brainstem

**FIGURE 4:**
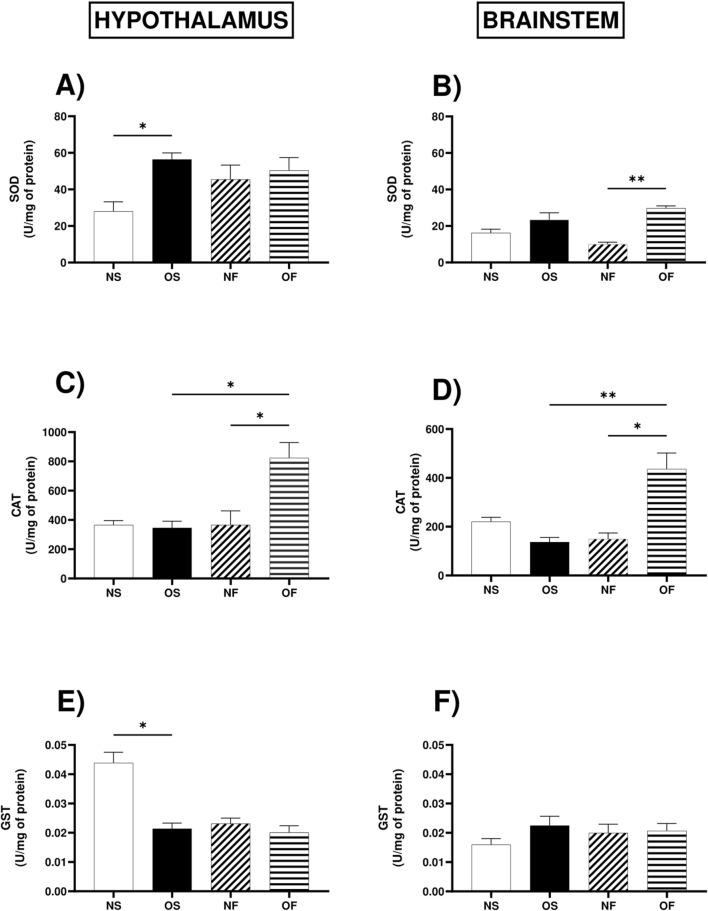
Antioxidant enzymatic activity in the hypothalamus and brainstem from female rats normo- or overfed during lactation period, treated of not with fluoxetine. **(A)** SOD activity in the hypothalamus; **(B)** SOD activity in the brainstem; **(C)** Catalase activity in the hypothalamus; **(D)** Catalase activity in the brainstem; **(E)** GST activity in the hypothalamus; **(F)** GST activity in the brainstem. The data were then expressed as medians, minimum and maximum scores. To compare the experimental groups, an analysis of variance was used using the Kruska-Wallis test with multiple comparisons and Dunn’s post-test. *p ≤ 0.05, **p ≤ 0.01, p ≤ 0.001, ****p ≤ 0.0001. The sample size was 6 animals per group.

The activity of the superoxide dismutase enzyme in the hypothalamus was higher in the OS female rats compared to the NS female rats, NS: 24.13 (17.38–43.95), (p = 0.032) (Figure 4A). In the brainstem, this enzyme showed more significant activity in the OF female rats than the NF female rats, NF: 8.91 (7.88–14.76) and OF: 30.60 (24.88–32.26) (p = 0.001) (Figure 4B).

In the hypothalamus, the activity of the enzyme catalase, serotonergic manipulation positively modulated the activity of this enzyme in the OF group compared to the NF rats, NF: 240.5 (190.2–743.5) and OF: 807.9 (571.2–1,092) and also positively modulated it about the group of female OS rats, OS: 324.2 (250.0–553.4) and OF: 807.9 (571.2–1,092), (p = 0.011; p = 0.032), respectively (Figure 4C). In the brainstem, catalase activity followed the same trend since serotonergic manipulation increased enzyme activity in OF female rats compared to NF, NF: 8.91 (7.88–14.76) and OF: 30.60 (24.88–32.26), with significance (p = 0.013), and there was also more significant activity in OF female rats compared to OS female rats, OS: 19.57 (14.76–36.65) and OF: 30.60 (24.88–32.26), with significance (p = 0.001) (Figure 4D).

The activity of the enzyme Glutathione-S-transferase in the hypothalamus was lower in OS female rats than NS female rats, NS: 0.047 (0.031–0.051) and OS: 0.019 (0.016–0.026), with significance (p = 0.035) (Figure 4E). In the brainstem, the activity of the GST enzyme showed no significant difference between the groups (Figure 4F).

### 3.8 Non-enzymatic antioxidant system in the hypothalamus and brainstem

**FIGURE 5:**
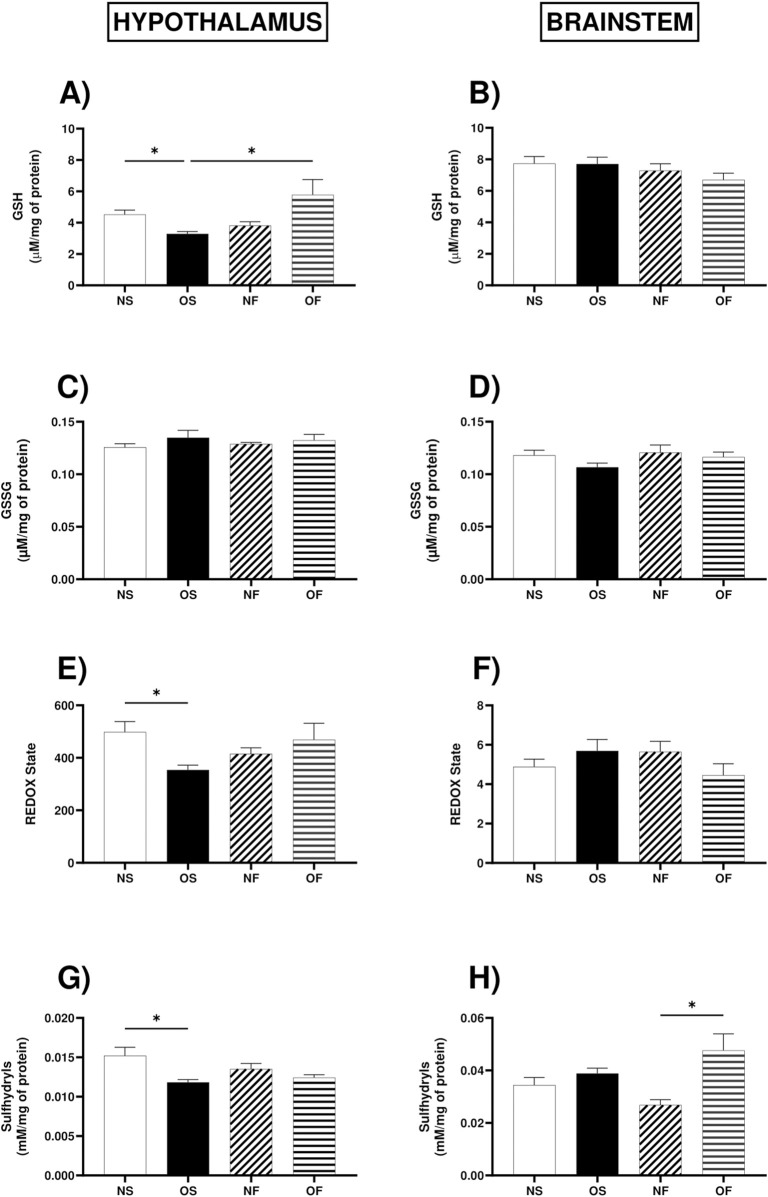
Non-enzymatic antioxidant system in the hypothalamus and brainstem from female rats normo- or overfed during lactation period, treated of not with fluoxetine. **(A)** GSH levels in the hypothalamus; **(B)** GSH levels in the brainstem; **(C)** GSSG levels in the hypothalamus; **(D)** GSSG levels in the brainstem; **(E)** REDOX state in the hypothalamus; **(F)** REDOX state in the brainstem; **(G)** Total thiol content in the hypothalamus; **(H)** Total thiol content in the brainstem. The data were then expressed as medians, minimum and maximum scores. To compare the experimental groups, an analysis of variance was used using the Kruska-Wallis test with multiple comparisons and Dunn’s post-test. *p ≤ 0.05, **p ≤ 0.01, p ≤ 0.001, ****p ≤ 0.0001. The sample size was 6 animals per group.

GSH in the hypothalamus showed higher levels in the OF female rats when compared to the OS female rats (OS: 3.24 (3.01–3.85) and OF: 5.84 (3.93–7.47)). OS was also lower when compared to female NS (NS: 4.28 (3.95–5.83) and OS: 3.24 (3.01–3.85)), with significance (p = 0.031 and p = 0.032, respectively) (Figure 5A). There was no difference between the brainstem groups (Figure 5B). As for oxidized glutathione content, there was no significant difference between the groups in the two tissues (Figures 5C,D). The REDOX state in the hypothalamus was affected by overnutrition in the OS female rats (NS: 454.8 (422.6–671.3) and OS: 335.6 (318.5–440.1)), with significant decrease in OS (p = 0.044) (Figure 5E). However, in the brainstem, it was not significant (Figure 5F). In the hypothalamus, the levels of total thiols were lower in the OS female rats than in the NS group of female rats (NS: 0.031 (0.028–0.042) and OS: 0.038 (0.033–0.044)), with significant decrease in OS (p = 0.017). In the brainstem, the content of thiols was significantly higher in OF female rats than in NF female rats (NF: 0.029 (0.020–0.031) and OF: 0.039 (0.036–0.064)), with significant increase in OF (p = 0.017) (Figures 5G,H).

### 3.9 BDNF expression in the hypothalamus and brainstem

**FIGURE 6:**
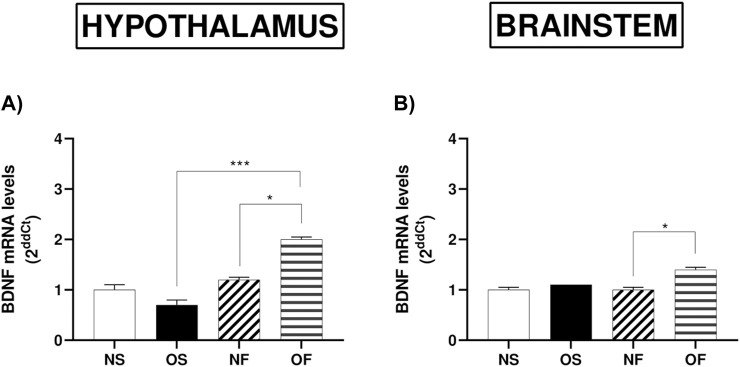
mRNA levels of BDNF in the hypothalamus and brainstem from female rats normo- or overfed during lactation period, treated of not with fluoxetine. **(A)** BDNF in the hypothalamus; **(B)** BDNF in the brainstem. Data presented as mean ± SEM. For comparison of groups by two-way ANOVA followed by Tukey’s test for multiple comparisons. *p ≤ 0.05, **p ≤ 0.01, p ≤ 0.001, ****p ≤ 0.0001. The sample size was 4 animals per group, measured in duplicate.

BDNF gene expression in the hypothalamus, we observed that OF animals had higher levels than in NF (p = 0.017). It was also higher in OF female rats than in OS (p = 0.0004) (Figure 6A). In the brainstem, we observed similar results, with the expression of this gene higher in OF female rats than in NF animals (p = 0.041) (Figure 6B).

## 4 Discussion

In this study, we evaluated the effects of maternal overnutrition during development in young females regarding body and behavioral changes, mitochondrial markers, oxidative balance, and expression of genes involved in neuroplasticity in the brainstem and hypothalamus. The association between excessive nutrient intake early in life and metabolic disorders is raising clinical concerns worldwide. Overweight or obesity, positively associated with an imbalance between food intake and energy expenditure, is considered a risk factor for several diseases, such as neurological conditions (Dikaiou et al., 2021). To date, experimental studies regarding the effects of overnutrition and obesity have only been conducted in males. However, in the present study, we evaluated the effects of overnutrition in females. We observed that overnutrition during the lactation period resulted in an obese phenotype in rats at 30 days of age. Among nutritional insult models, Plagemann mimicked overnutrition in experimental groups by reducing litter size to investigate the potential long-term consequences of postnatal overfeeding on offspring (Plagemann et al., 1992a; Plagemann et al., 1992b). In this model, post-weaning, offspring of the reduced litter usually have a higher body weight than the control group (da Silva et al., 2019; Braz et al., 2020a). Furthermore, increased availability of maternal milk may lead to increased food consumption and, consequently, overweight (Gluckman et al., 2005; Braz et al., 2020b; Liu et al., 2013). These observations are supported by the fact that the newborn rat does not have control over food intake until the 14th or 16th day of postnatal life (McMillen et al., 2005) because hypothalamic control in early postnatal life is not fully structured yet (Pozzo Miller and Aoki, 1992). Similar to what has been observed previously, overnutrition in female rats also increased body weight, specifically on the 14th day. These findings are in line with Braz et al. (2020a) and de Andrade Silva et al. (2021), Gluckman et al. (2005), Braz et al. (2020b), whose increased body weight is related to the ample supply of maternal milk and the consequent lack of satiety signaling in male rats (Liu et al., 2013).

The Lee index serves as another indicator of overweight, specifically designed to assess obesity in rats and measure its severity (Plagemann et al., 1992b). In our research, we noted an increase in the Lee index among obese models, and unexpectedly, fluoxetine treatment was found to lower this index. Currently, various strategies are being implemented for body weight management, including medications from the Selective Serotonin Reuptake Inhibitors (SSRIs) category, with fluoxetine (commonly known as Prozac) being one of the most widely used (Braz et al., 2020a). Experimental studies on overfed male rats have shown that serotonin manipulation led to weight loss compared to controls (Gluckman et al., 2005; Sui and Pasco, 2020; Plagemann et al., 1992a). Additionally, it is important to highlight that fluoxetine administration in overfed rats also resulted in reduced glycemic and lipid levels in this study. Clinical studies have similarly indicated that fluoxetine positively impacts glycemic and weight control in obese individuals (Braz et al., 2020b), suggesting that the medication not only aids in reducing body weight but also enhances blood profiles.

In the perspective to understand the result with body weight and Lee index we evaluate the eating preference. Eating preference of high palatable foods may be associated with neurobehavioral and metabolic changes related to obesity (Ali et al., 2018), showing the complexity of interactions between the central nervous system and appetite regulation. In our study, in overfed female rats, we observed a significant increase in consumption of palatable foods, such as cookies, compared to non-obese animals. In addition, obese females also consumed more standard feed compared to non-obese animals, showing hyperphagic behavior associated with overnutrition early in life. In addition, we observed that obese rats eat more overall compared to non-obese rats. These results follow the data from Silva et al., (2021), (da Silva et al. (2019), Gluckman et al. (2005), suggesting that preference for palatable foods and increased total food consumption may be a phenomenon observed in both sexes, although previous studies have only been conducted in male rats. This indicates a possible relationship between obesity and response to consumption of highly palatable foods, regardless of sex (Stice et al., 2009). It is worth noting that one of the actions of fluoxetine, through the modulation of serotonin levels, is to control appetite, which can cause a reduction in food intake, decrease in Lee index, glucose levels, and cholesterol, a response observed in overnourished rats, confirming the role of fluoxetine as an important appetite modulator regardless of the animal’s gender.

As mentioned earlier, it is already known that overnutrition and obesity induce metabolic changes that may be related to numerous chronic diseases (Ahmed et al., 2021). The primary regulatory and controlling site of cellular metabolism is the mitochondria, as they play a fundamental role in the biology of most eukaryotic cells, being involved in the regulation of metabolism, control of intracellular calcium homeostasis, initiation of inflammatory reactions, and control of various pathways influencing cellular life and death (Casanova et al., 2023). Studies have shown that mitochondrial impairment due to obesity is closely associated with progressive remodeling or organ failure (Hadjispyrou et al., 2023; Chen et al., 2023).

In the hypothalamus of the groups studied, we observed no differences in the levels of the coenzymes NAD+ and NADH concerning mitochondrial function. However, in the brainstem, the NADH levels were found to be lower in overnourished animals. Interestingly, manipulating the serotonergic system led to an increase in NADH levels in these animals. In the brainstem, the NAD+/NADH ratio was diminished in overnourished animals treated with fluoxetine compared to both overnourished and normally nourished females receiving fluoxetine. This suggests an increase in metabolic activity related to substrate oxidation, as the NAD+/NADH ratio is crucial for the proper functioning of cellular redox reactions (Ahmed et al., 2021). In relation to mitochondrial function, we assessed the activity of citrate synthase, a key enzyme in the oxidative system that initiates the reactions of the citric acid cycle; our findings indicated that overnutrition adversely affected the activity of this enzyme in the hypothalamus. Conversely, in overnourished females treated with fluoxetine, enzyme activity in the brainstem was restored compared to their controls. These findings highlight a varied response of citrate synthase enzyme activity to overnutrition and serotonergic manipulation across different brain regions. The reduced activity of this enzyme in overnourished animals may result from metabolic and bioenergetic alterations associated with obesity (Casanova et al., 2023). This condition is frequently linked with metabolic dysfunctions, including insulin resistance and disruptions in lipid and carbohydrate metabolism, which can influence energy metabolism (Hadjispyrou et al., 2023). As previously mentioned, we noted a decrease in citrate synthase enzyme activity in normally nourished animals treated with fluoxetine, which contrasts with the findings of Silva et al. (2021) in the prefrontal cortex of male rats at 22 days (Chen et al., 2023). It remains unclear whether this decrease is influenced by tissue type, age, or sex (Gluckman et al., 2005; Collin, 2019). Nonetheless, it is established that serotonergic system manipulation may not yield positive effects in normally fed rats, given the delicate balance in neurotransmitter regulation and metabolic homeostasis (Aksenov and Markesbery, 2001; Hissin and Hilf, 1976; Collin, 2019; Palmisano et al., 2018). During obesity, increased levels of reactive oxygen species (ROS) are commonly observed (Plagemann et al., 1992a). However, it remains unclear whether these increased levels are a cause or a consequence of mitochondrial dysfunction and the cellular metabolism imbalance associated with obesity (Clarkson and Thompson, 2000). ROS are natural byproducts of cellular metabolism, but when produced in excess, they can lead to oxidative stress, damaging biomolecules and compromising mitochondrial function (de Andrade Silva et al., 2023). The rise in ROS production in the hypothalamus of overnourished rats may be primarily attributed to obesity itself. Conversely, in normally nourished animals treated with fluoxetine, the increase in ROS in both the hypothalamus and brainstem can be linked to the pharmacological effects of the drug. Another potential mechanism for the heightened ROS levels in these tissues of normally nourished rats treated with fluoxetine could be a reduction in citrate synthase activity, as mitochondrial dysfunction can result in cellular damage (Stice et al., 2009; Allegra et al., 2023). Despite this possible explanation, we cannot dismiss the likelihood of regulatory mechanisms at play; a decrease in citrate synthase expression may lead to excessive superoxide formation and cellular apoptosis (Strehlow et al., 2003). Overall, it can be suggested that obesity—regardless of species, gender, or age—is associated with increased levels of ROS. Further studies are needed to better understand the specific mechanisms underlying this molecular and cellular disorder.

The main ROS are superoxide anion (O_2_-), hydrogen peroxide (H_2_O_2_), and the hydroxyl radical (-OH), which can induce lipid, protein, and DNA oxidation, leading to what is known as oxidative stress (Collin, 2019). Experimentally, lipid peroxidation can be assessed by measuring the levels of malondialdehyde; in our animals, we did not observe a difference in the hypothalamus of overnourished rats; however, serotonergic manipulation reduced lipid damage in overnourished females treated with fluoxetine compared to normal-nourished females. In the brainstem, there was no difference between the groups. Overall, there are sex differences in susceptibility to lipid oxidation. Women tend to have higher levels of endogenous antioxidants and hormonal interference, which may provide additional protection against lipid oxidation (Palmisano et al., 2018), justifying our data mainly in the brainstem. In addition to the analyses of lipid peroxidation, we evaluated protein oxidation, whose results in both tissues indicate an increase in protein damage in overnourished female rats; however, fluoxetine treatment in this group contributed to a reduction in protein oxidation levels compared to both control groups. It is essential to consider that, despite the antioxidant protection conferred by estrogen and other physicochemical properties that can reduce lipid oxidation, women may still suffer oxidative damage to proteins or DNA due to various other factors, such as environmental stress. These factors may include specific conditions such as chronic inflammation, nutritional excesses, or exposure to exogenous oxidants, which can challenge the protective efficacy of estrogen and increase protein oxidation (Clarkson and Thompson, 2000).

Emerging studies suggest that estrogen significantly boosts the expression of antioxidant enzymes, enhancing the antioxidant defense system (Donovan and Tecott, 2013; Sandrini et al., 2018). In our research, we observed distinct variations in superoxide dismutase (SOD) activity across tissues. While no significant differences were found in the brainstem between obese and control groups, a notable increase in SOD activity was recorded in the fluoxetine-treated obese group. In the hypothalamus, SOD activity increased with obesity, as this enzyme converts superoxide into hydrogen peroxide and oxygen. However, it’s essential that this increase in SOD is matched by elevated catalase activity, which breaks down hydrogen peroxide (Li et al., 2022; Taha et al., 2022); otherwise, SOD can act as a “double-edged sword.” The rise in SOD activity in overnourished rats may be linked to increased protein peroxidation, emphasizing the need for further research on additional enzymes. Importantly, serotonergic manipulation increased catalase activity in overnourished female rats in both tissues. Our findings align with studies on overnourished males, indicating effective stimulation of catalase activity in the hippocampus at 22 days (Palmisano et al., 2018), and in the hypothalamus and brainstem at 90 days (Collin, 2019). Additionally, the reduced activity of glutathione S-transferase (GST) in the hypothalamus of overnourished rats reinforces the notion that the negative effects of overnutrition during development are independent of gender and age, underscoring the urgency for continued investigation in this area.

The antioxidant system is composed of enzymes and non-enzymatic molecules that can neutralize ROS, thus minimizing the deleterious action of these reactive molecules. Among the non-enzymatic antioxidant molecules, we evaluated reduced glutathione (GSH) levels and total thiols (-SH). Regarding GSH, we did not observe a difference in the brainstem; however, in the hypothalamus, we observed that in overnourished females, there is a reduction, whereas, in overnourished female rats, treatment with fluoxetine led to a significant increase in GSH levels. Accompanied with decrease in GSH we observed and significant decrease in REDOX status in obese group; corroborating our results with previous data in literature were showed that overnutrition decrease REDOX status in male with 22 days (de Andrade Silva et al., 2023). It is essential to highlight that the response to nutritional or pharmacological insult may be tissue-dependent; however, overall antioxidant capacity is higher in females (Allegra et al., 2023); mainly, there are discussions that estrogen, in females, may more efficiently modulate the enzymatic defense system (Strehlow et al., 2003; Bellanti et al., 2013). Additionally, at least in male rats, serotonergic manipulation increased antioxidant capacity (Braz et al., 2016). As for the total thiols (-SH) content in the hypothalamus, we observed that in overnourished animals, there was a reduction, indicating a decrease in antioxidant capacity under these overfeeding conditions and corroborating with previous studies (Çakırca et al., 2022). In the brainstem, we did not observe a difference in overnourished females, but in overnourished females treated with fluoxetine, the levels of this antioxidant defense marker were elevated. These differences in antioxidant responses between the hypothalamus and brainstem may be due to the different functions and metabolic demands of these brain regions, as well as the complexity of the serotonergic system and its interactions with other neuromodulator systems and metabolic pathways (Donovan and Tecott, 2013).

Previous studies suggest that a high-fat diet can damage neurotransmitter pathways, such as the serotonergic system, and alter BDNF levels due to various factors (Sandrini et al., 2018). BDNF is an essential protein that develops, grows, and maintains neurons in the central nervous system (Li et al., 2022). Previous data in literature demonstrated that deletion of the BDNF gene lead to severe obesity and insatiable appetite, in addition studies with overfed and fluoxetine, showed that FX-treatment increases BDNF levels, strengthening the idea that the serotonin modulation act as an important BDNF regulator and the increase in its levels may activate the path to decrease body weight through induction of satiety (Chen et al., 2023). In addition, one study conducted in overnourished male rat, demonstrated that FX-treatment, induced positive response on oxidative balance in hippocampus in parallel to the increase in the BDNF mRNA levels. Both previous studies corroborate with our data, since, we observed alterations in overnourished female rats treated with fluoxetine, where we found an increase in the expression of this gene, which may be related to the regulation of feeding behavior, since in overnourished females treated with fluoxetine, we observed greater satiety compared to overnourished females treated with saline, corroborating with previous studies (de Andrade Silva et al., 2023; Taha et al., 2022).

## 5 Conclusion

From all the data obtained with the present study, we can conclude that maternal overnutrition in female rat’s results in phenotypic, behavioral, blood, cellular, and molecular alterations, causing significant damage to essential biomolecules, as well as impairing the activity of antioxidant enzymes and oxidative metabolism enzyme. However, serotonergic manipulation has shown to be beneficial in overnourished female rats by improving the efficacy of antioxidant system activity, enhancing oxidative metabolism enzyme activity (i.e., citrate synthase), increasing the expression of genes associated with neurogenesis, and consequently reducing oxidative stress markers. While mitochondrial dysfunction linked to oxidative stress is reported as an underlying mechanism of overnutrition and obesity, the exact link between overnutrition, mitochondrial dysfunction, and oxidative stress in both sex is poorly understood and requires further investigation.

## 6 Limitations

Although our study brings a new perspective for intervention and shows that even though females are naturally less susceptible to oxidative stress, obesity at an early age overlaps with the natural effects of female hormones, some limitations should be acknowledged. First, potential litter effects were not accounted for in the experimental design, which may influence the variability of metabolic and behavioral outcomes. Second, while we explored sex-specific differences in oxidative stress susceptibility, the study did not fully investigate potential interactions between sex hormones and serotonergic pathways in the context of early-life obesity. Third, the sample size, though sufficient for detecting major effects, may limit the statistical power to identify subtler interactions or sex-dependent responses. Additionally, since we used a pharmacological agent, we need to verify whether other interventions that enhance the release and action of serotonin but with a lower risk of side effects could also act as a therapeutic agent, decreasing the levels of oxidative stress and food consumption. With the data obtained in this study, more questions opened that we hope to answer soon, thus evaluating mitochondrial bioenergetics, the activity of mitochondrial complexes, calcium signaling, generalized and local inflammatory processes, and protein levels of neurotrophic, among other analyses, to better understand how childhood overnutrition linked to early obesity damages brain areas connected to behavior.

## Data Availability

The original contributions presented in the study are included in the article/supplementary material, further inquiries can be directed to the corresponding authors.
