# Supplementary material for: Serotoninergic modulation in the brainstem and hypothalamus of female overnourished rats: impact on mitochondrial markers, oxidative stress and BDNF mRNA levels
Source: Front Mol Biosci. 2025 May 16;12:1564061. doi: 10.3389/fmolb.2025.1564061 (PMC12123090; doi:10.3389/fmolb.2025.1564061)
Supplement: Supplementary file 1 [file Table1.docx]

**Suplemmentary Table 1*.*** Effect of Fluoxetine on Metabolic Parameters, Mitochondrial Function, Oxidative Stress and Gene Expression in the Brainstem.

| **Variables** | **Normofed + Fluoxetine** | **Overfed + Fluoxetine** | ***g*** | ***Effect Size Classification*** |
| --- | --- | --- | --- | --- |
| **Metabolic Parameters**  Body weight at 30 days | 59.67 ± 3.64 | 75.88 ± 5.98 | **3.32** | **Very Large** |
| Lee Index  Cook Preference  Labina Preference  Total Consumption (Cook+Labina) **Biochemical Parameters**  HDL  Triglycerides  Total Cholesterol  Glucose | 296.8 ± 46.3  2.20 ± 0.19  3.20 ± 0.30  5.40 ± 0.35  87.58 ± 7.61  71.11 ± 11.7  65.16 ± 1.79  164.8 ± 17.4 | 290.0 ± 3.74  2.51 ± 0.04  1.20 ± 0.12  3.71 ± 0.09  78.89 ± 2.24  67.78 ± 4.77  55.41 ± 5.23  144.9 ± 8.55 | 0.20  **2.05**  -**8.10**  **6.03**  **-1.46**  -0.37  **-2.49**  **-1.45** | Ignored  **Very Large**  **Very Large**  **Very Large**  **Very Large**  Small  **Very Large**  **Very Large** |
| **Mitochondrial markers**  Citrate Synthase  NAD^+^  NADH  NAD^+^/NADH  DCF | 0.53 ± 0.07  3.09 ± 0.94  0.55 ± 0.20  5.30 ± 1.08  0.97 ± 0.02 | 1.74 ± 0.36  3.69 ± 0.60  0.73 ± 0.25  3.82 ± 0.24  0.88 ± 0.02 | **4.66**  0.73  **0.81**  **-1.74**  **-4.50** | **Very Large**  Moderate  **Large**  **Very Large**  **Very Large** |
| **Oxidative Balance** |  |  |  |  |
| TBARS | 4.975 ± 1.62 | 6.555 ± 0.975 | **1.18** | **Large** |
| Carbonyls | 22.85 ± 1.84 | 7.99 ± 2.109 | **-7.50** | **Very Large** |
| Superoxide Dismutase | 10.02 ± 2.72 | 29.79 ± 2.83 | **7.13** | **Very Large** |
| Catalase | 149.1 ± 62.0 | 436.3 ± 173.0 | **2.13** | **Very Large** |
| Glutathione S transferase | 0.01 ± 0.007 | 0.02 ± 0.005 | **1.61** | **Very Large** |
| Reduced Glutathione (GSH) | 7.872 ± 1.06 | 6.697 ± 1.03 | -0.56 | Moderate |
| Oxidized Glutathione (GSSG) | 0.120 ± 0.01 | 0.116 ± 0.01 | -0.40 | Small |
| REDOX State (GSH/GSSG) | 5.647 ± 1.29 | 4.454 ± 1.29 | **-0.92** | **Large** |
| Sulfhydryl’s | 0.02 ± 0.004 | 0.04 ± 0.014 | **1.94** | **Very Large** |
| **Gene Expression**  BDNF | 1.000 ± 0.100 | 1.400 ± 0.100 | **4.00** | **Very Large** |

BDNF: Brain-Derived Neurotrophic Factor; DCF: dihydrodichlorofluorescein; NAD+: Nicotinamide Adenine Dinucleotide; NADH: Nicotinamide Adenine Dinucleotide

Reduced; TBARS: thiobarbituric acid reactive substances.

**Suplemmentary Table 2*.*** Effect of Fluoxetine on Mitochondrial Function, Oxidative Stress and Gene Expression in the Hypothalamus.

| **Variables** | **Normofed + Fluoxetine** | **Overfed + Fluoxetine** | ***g*** | ***Effect Size Classification*** |
| --- | --- | --- | --- | --- |
| **Mitochondrial markers**  Citrate Synthase  NAD^+^  NADH  NAD^+^/NADH  DCF | 0.83 ± 0.34  1.96 ± 0.81  0.34 ± 0.21  3.14 ± 1.08  0.93 ± 0.02 | 1.31 ± 0.37  1.86 ± 0.47  0.44 ± 0.20  4.32 ± 1.13  0.86 ± 0.04 | **1.33**  -0.13  **0.81**  **5.63**  **-2.05** | **Very Large**  Ignored  **Large**  **Very Large**  **Very Large** |
| **Oxidative Balance** |  |  |  |  |
| TBARS | 13.10 ± 2.22 | 9.64 ± 1.15 | **-2.05** | **Very Large** |
| Carbonyls | 26.87 ± 4.89 | 8.43 ± 1.85 | **-4.98** | **Very Large** |
| Superoxide Dismutase | 45.41 ± 17.6 | 50.36 ± 15.8 | 0.29 | Small |
| Catalase | 365.4 ± 237.2 | 711.8 ± 300.5 | **1.18** | **Very Large** |
| Glutathione S transferase | 0.023 ± 0.004 | 0.020 ± 0.006 | -0.58 | Moderate |
| Reduced Glutathione (GSH) | 3.811 ± 0.625 | 5.324 ±1.958 | **1.10** | **Large** |
| Oxidized Glutathione (GSSG) | 0.128 ± 0.003 | 0.132 ± 0.015 | 0.34 | Small |
| REDOX State (GSH/GSSG) | 415.1 ± 57.11 | 469.2 ± 177.6 | 0.38 | Small |
| Sulfhydryl’s | 0.013 ± 0.001 | 0.012 ± 0.0009 | **-1.05** | **Large** |
| **Gene Expression**  BDNF | 1.200 ± 0.100 | 2.000 ± 0.100 | **8.00** | **Very Large** |

BDNF: Brain-Derived Neurotrophic Factor; DCF: dihydrodichlorofluorescein; NAD+: Nicotinamide Adenine Dinucleotide; NADH: Nicotinamide Adenine Dinucleotide

Reduced; TBARS: thiobarbituric acid reactive substances.
